# Supplementary material for: Rural-urban disparity in induced abortion in Ghana: A multivariate non-linear decomposition analysis of the Ghana Maternal Health Survey
Source: PLoS One. 2025 Oct 15;20(10):e0334024. doi: 10.1371/journal.pone.0334024 (PMC12527179; doi:10.1371/journal.pone.0334024)
Supplement: Attachment — Submitted filename: PONE-D-24-59727 (1).pdf [file pone.0334024.s002.pdf]

PLOS ONE

Rural-urban disparity in induced abortion in Ghana: a multivariate non-linear decomposition analysis of Ghana Maternal Health Survey

--Manuscript Draft--

**Manuscript Number:**	PONE-D-24-59727
**Article Type:**	Research Article
**Full Title:**	Rural-urban disparity in induced abortion in Ghana: a multivariate non-linear decomposition analysis of Ghana Maternal Health Survey
**Short Title:**	Rural-urban disparity in induced abortion in Ghana
**Corresponding Author:**	Sarah Asaah Owusu-Kwankye, Masters<br>University of Ghana<br>Accra, Greater Accra Region GHANA
**Keywords:**	induced abortion; rural; urban; Disparity; GMHS; Ghana
**Abstract:**	Globally, 73.3 million induced abortions were recorded between 2015 and 2019. There are significant disparities in induced abortions across the rural-urban divide that necessitates targeted policies. In this study, we decomposed the rural-urban disparities in induced abortion in Ghana. Data for the study was extracted from the most recent 2017 Ghana Maternal Health Survey. The sample for this study comprised of women who have ever been pregnant resulting in a weighted sample of 18,140. A multivariate non-linear decomposition model was employed to decompose the rural-urban disparities in induced abortion. The results were presented using coefficients and percentages. The results were presented using coefficients and percentages. The proportion of women who have had induced abortions in their lifetime in the study was 27.1%. Induced abortion was higher in urban areas (34.1%) than in rural areas (19.4%). Approximately 55% of the rural-urban disparities in induced abortion were attributable to differences in women's socio-demographic and obstetric characteristics. Hence, if women’s socio-demographic and obstetric characteristics were equalled, the rural-urban disparity in induced abortion would be decreased. Region of residence (25.4%), education (16.6%), and parity (9.4%) explained approximately 51% of the rural-urban inequality in induced abortion. This study shows significant rural-urban disparities in induced abortion, with the disparities being attributable to the differences in socio-demographic and obstetrics characteristics: region of residence, education, and parity. Policymakers could focus and work on intensifying sexual and reproductive health educational messages, particularly, among women residing in the middle and southern ecological zone of Ghana, and also targeting the educated.
**Order of Authors:**	Isaac Yeboah, Doctorate degree
	Martin Wiredu Agyekum, Doctorate degree
	Duah Dwomoh, Doctorate degree
	Desmond Klu, Doctorate degree
	Mary Naana Essiaw, Doctorate degree
	Andrew Conduah, Masters degree
	Sarah Asaah Owusu-Kwankye, Masters degree
**Additional Information:**	
**Question**	**Response**
**Financial Disclosure**<br><br>Enter a financial disclosure statement that describes the sources of funding for the work included in this submission. Review the submission guidelines for detailed	The author(s) received no specific funding for this work.

Powered by Editorial Manager® and ProduXion Manager® from Aries Systems Corporation

requirements. View published research articles from *PLOS ONE* for specific examples.<br><br>This statement is required for submission and **will appear in the published article** if the submission is accepted. Please make sure it is accurate.<br><br>**Funded studies**<br>Enter a statement with the following details:<br>• Initials of the authors who received each award<br>• Grant numbers awarded to each author<br>• The full name of each funder<br>• URL of each funder website<br>• Did the sponsors or funders play any role in the study design, data collection and analysis, decision to publish, or preparation of the manuscript?<br><br>Did you receive funding for this work?	
**Competing Interests**<br><br>Use the instructions below to enter a competing interest statement for this submission. On behalf of all authors, disclose any competing interests that could be perceived to bias this work—acknowledging all financial support and any other relevant financial or non-financial competing interests.<br><br>This statement is required for submission and **will appear in the published article** if the submission is accepted. Please make sure it is accurate and that any funding sources listed in your Funding Information later in the submission form are also declared in your Financial Disclosure statement.<br><br>View published research articles from *PLOS ONE* for specific examples.	The authors have declared that no competing interests exist

**NO authors have competing interests**<br><br>Enter: *The authors have declared that no competing interests exist.*<br><br>**Authors with competing interests**<br><br>Enter competing interest details beginning with this statement:<br><br>*I have read the journal's policy and the authors of this manuscript have the following competing interests: [insert competing interests here]*<br><br>* typeset	
**Ethics Statement**<br><br>Enter an ethics statement for this submission. This statement is required if the study involved:<br><br>• Human participants<br>• Human specimens or tissue<br>• Vertebrate animals or cephalopods<br>• Vertebrate embryos or tissues<br>• Field research<br><br>Write "N/A" if the submission does not require an ethics statement.<br><br>General guidance is provided below. Consult the submission guidelines for detailed instructions. **Make sure that all information entered here is included in the Methods section of the manuscript.**	The 2017 Ghana Maternal Health Survey was conducted under the scientific guidance of the Ghana Statistical Service, Ghana Health Service (GHS), and Noguchi Memorial Institute for Medical Research (NMIMR). ICF International approved the survey and provided technical assistance. Written Informed consent was obtained from all the respondents before the commencement of interviews with each interviewer.

Powered by Editorial Manager® and ProduXion Manager® from Aries Systems Corporation

**Format for specific study types**<br><br>**Human Subject Research (involving human participants and/or tissue)**<br>• Give the name of the institutional review board or ethics committee that approved the study<br>• Include the approval number and/or a statement indicating approval of this research<br>• Indicate the form of consent obtained (written/oral) or the reason that consent was not obtained (e.g. the data were analyzed anonymously)<br><br>**Animal Research (involving vertebrate animals, embryos or tissues)**<br>• Provide the name of the Institutional Animal Care and Use Committee (IACUC) or other relevant ethics board that reviewed the study protocol, and indicate whether they approved this research or granted a formal waiver of ethical approval<br>• Include an approval number if one was obtained<br>• If the study involved *non-human primates*, add *additional details* about animal welfare and steps taken to ameliorate suffering<br>• If anesthesia, euthanasia, or any kind of animal sacrifice is part of the study, include briefly which substances and/or methods were applied<br><br>**Field Research**<br><br>Include the following details if this study involves the collection of plant, animal, or other materials from a natural setting:<br>• Field permit number<br>• Name of the institution or relevant body that granted permission	
**Data Availability**<br><br>Authors are required to make all data underlying the findings described fully available, without restriction, and from the time of publication. PLOS allows rare exceptions to address legal and ethical concerns. See the PLOS Data Policy and FAQ for detailed information.	Yes - all data are fully available without restriction

Powered by Editorial Manager® and ProduXion Manager® from Aries Systems Corporation

A Data Availability Statement describing where the data can be found is required at submission. Your answers to this question constitute the Data Availability Statement and **will be published in the article**, if accepted.<br><br>**Important:** Stating 'data available on request from the author' is not sufficient. If your data are only available upon request, select 'No' for the first question and explain your exceptional situation in the text box.<br><br>Do the authors confirm that all data underlying the findings described in their manuscript are fully available without restriction?	
**Describe where the data may be found in full sentences. If you are copying our sample text, replace any instances of XXX with the appropriate details.**<br><br>• If the data are **held or will be held in a public repository**, include URLs, accession numbers or DOIs. If this information will only be available after acceptance, indicate this by ticking the box below. For example: *All XXX files are available from the XXX database (accession number(s) XXX, XXX.*).<br>• If the data are all contained **within the manuscript and/or Supporting Information files**, enter the following: *All relevant data are within the manuscript and its Supporting Information files.*<br>• If neither of these applies but you are able to provide **details of access elsewhere**, with or without limitations, please do so. For example:<br><br>*Data cannot be shared publicly because of [XXX]. Data are available from the XXX Institutional Data Access / Ethics Committee (contact via XXX) for researchers who meet the criteria for access to confidential data.*<br><br>*The data underlying the results presented in the study are available from (include the name of the third party*	The datasets generated and analyzed for this study are available in the MEASURE DHS database at the repository; https://dhsprogram.com/data/dataset/Ghana_Special_2017.cfm?flag=1

Powered by Editorial Manager® and ProduXion Manager® from Aries Systems Corporation

and contact information or URL).<br>• This text is appropriate if the data are owned by a third party and authors do not have permission to share the data.<br><br>* typeset	
Additional data availability information:	Tick here if the URLs/accession numbers/DOIs will be available only after acceptance of the manuscript for publication so that we can ensure their inclusion before publication.

Powered by Editorial Manager® and ProduXion Manager® from Aries Systems Corporation

Manuscript Click here to access/download;Manuscript;Manuscript_docx.docx

Title Page

Author Information

Author Names

Isaac Yeboah[1] Martin Wiredu Agyekum[2] Duah Dwomoh[3] Desmond Klu[4] Mary Naana Essiaw[1] Andrew Kweku Conduah[1] Sarah Asaah Owusu-Kwankye[5*]

*Corresponding author – Sarah Asaah Owusu-Kwankye

ORCID identifier - 0009-0000-5974-0853, Email address – sarahkwankye026@gmail.com

Author Affiliations

[1] Institute of Work, Employment and Society, University of Professional Studies, Accra, Ghana.

[2] Institute for Educational Research and Innovation Studies (IERIS), University of Education, Winneba, Ghana.

[3]Department of Biostatistics, School of Public Health, University of Ghana, Legon, Ghana

[4]Institute of Heath Research (IHR), University of Health and Allied Sciences, Ho, Ghana

[5]Regional Institute for Population Studies, University of Ghana, Legon, Ghana

Rural-urban disparity in induced abortion in Ghana: a multivariate non-linear decomposition analysis of Ghana Maternal Health Survey

Abstract

Globally, 73.3 million induced abortions were recorded between 2015 and 2019. There are significant disparities in induced abortions across the rural-urban divide that necessitates targeted policies. In this study, we decomposed the rural-urban disparities in induced abortion in Ghana. Data for the study was extracted from the most recent 2017 Ghana Maternal Health Survey. The sample for this study comprised of women who have ever been pregnant resulting in a weighted sample of 18,140. A multivariate non-linear decomposition model was employed to decompose the rural-urban disparities in induced abortion. The results were presented using coefficients and percentages. The results were presented using coefficients and percentages. The proportion of women who have had induced abortions in their lifetime in the study was 27.1%. Induced abortion was higher in urban areas (34.1%) than in rural areas (19.4%). Approximately 55% of the rural-urban disparities in induced abortion were attributable to differences in women's socio-demographic and obstetric characteristics. Hence, if women’s socio-demographic and obstetric characteristics were equalled, the rural-urban disparity in induced abortion would be decreased. Region of residence (25.4%), education (16.6%), and parity (9.4%) explained approximately 51% of the rural-urban inequality in induced abortion. This study shows significant rural-urban disparities in induced abortion, with the disparities being attributable to the differences in socio-demographic and obstetrics characteristics: region of residence, education, and parity. Policymakers could focus and work on intensifying sexual and reproductive health educational messages, particularly, among women residing in the middle and southern ecological zone of Ghana, and also targeting the educated.

Keywords: induced abortion, rural, urban, disparity, GMHS, Ghana

Introduction

Since the 1966 International Covenant on Economic, Social, and Cultural Rights, the rights of women to access maternal and reproductive health have been on the priority agenda of the international community [1]. Subsequently, the 1994 International Conference on Population and Development (ICPD) established the importance of women's autonomy and their rights to access abortion services [2]. Beyond these actions, there are currently the sustainable development goals (SDGs) which target 3.1 (i.e., reduce the global maternal mortality ratio to less than 70 per 100 000 livebirths) and 3.7 (i.e., ensure universal access to sexual and reproductive healthcare services by 2030) inherently have implications on induced abortion worldwide [3]. Despite these policy frameworks, the incidence of induced abortion remains a significant public health concern as most of these abortions are categorized as unsafe. That is, "an abortion that is carried out either by a person lacking the necessary skills or in an environment that does not conform to minimal medical standards, or both" [4].

Available evidence indicates that nearly 73.3 million induced abortions were reported across the globe yearly between 2015 and 2019 [5]. Out of this figure, 45% of these abortions were categorized as unsafe with 97% of all unsafe abortions occurring in low-and-middle-income countries [1]. Regarding adverse outcomes of induced abortion, 62% of all induced abortion-related mortalities are reported in Africa [6]. In Ghana, 57.5% of induced abortions in the country were provided by unskilled providers, thereby making the procedure unsafe [7]. Nevertheless, safe induced abortion is considered a human right of every woman [8]. Hence, accessibility to safe induced abortion reduces the incidence of unsafe abortions and its concomitant health outcomes.

There is a preponderance of evidence showing the factors that influence women to have an induced abortion. Among the factors identified in previous studies include non-use of contraceptives [9], educational attainment level [7], gender preferences [10], marital status and region of residence [11], unmet need for contraception [12], wealth status and parity [13]. Beyond these factors, it is undisputed that place of residence plays a critical role in health service utilization, including the decision to have an induced abortion. Studies conducted in China [14] and India [15] have shown that there are significant disparities in induced abortions across the rural-urban divide. For instance, Rahaman et al. [15] study showed women from poorer households were significantly more likely to have an unsafe induced abortion when they lived in rural areas than when they

resided in urban areas. An understanding of the rural-urban disparities is imperative to identify the factors that influence induced abortions exclusively in either rural or urban areas or those that are uniform across both places of residence. This would ensure the development of targeted policies and interventions. Nevertheless, no study in Ghana has disaggregated the associated factors of induced abortions across the dimension of place of residence. This situation suggests a significant knowledge gap in the current discourse of induced abortion in Ghana. The current study, therefore, seeks to examine the rural-urban disparities in induced abortion in Ghana.

Theoretical Framework

This study is underpinned by the social learning theory. The social learning theory proposes that the environment is a major force in human behavior in that human behavior is influenced by observation, modeling, and imitations [16-17]. An individual learns behavior by observing others in a social setting such as family, friends, teachers, neighbors, and church groups. Individuals assimilate and imitate that behavior when they are associated with rewards or positive experiences. Bandura asserts that reinforcement can account for social learning. Reinforcement is when an individual is aware of prior experience consequences. If the consequence of abortion is positive there is the likelihood of its occurrence and vice versa.

Methods

Data source and study design

Data for the study was extracted from the most recent Ghana Maternal Health Survey (GMHS) conducted in 2017. We used the women's individual recode files. The GMHS is a nationally representative cross-sectional survey that uses a two-stage cluster sampling method. The study was restricted to women who have ever been pregnant. This is because pregnancy exposes an individual to the likelihood of induced abortion.

Outcome variable

Induced abortion was the outcome variable in this study. Women were asked 'How many pregnancies have ended this way (abortion) in your lifetime?' The response categories yielded responses such as none (0), 1, 2, or more. Those with none were coded as "0=no" and those who have had 1 or more were coded as "1=yes".

Primary exposure

The main explanatory variable was place of residence. The responses for this were “rural” and “urban”.

Potential confounders

The potential confounders were selected based on their association with induced abortion in literature. The covariates include age, region of residence, educational attainment, marital status, ethnicity, religion, current contraceptive use, age at first sex, knowledge of ovulation, parity, knowledge of abortion legality, and migration status. The categories of each of the variables are shown in Table 1.

Statistical analyses

Data for the study were analyzed using Stata version 16 (StataCorp, College Station, Texas, USA). First, the distribution of induced abortion across explanatory variables and covariates was examined using the chi-square test. The results were further disaggregated by place of residence. Third, multivariable binary logistic regression analysis was carried out to explore the predictors of induced abortion by place of residence. Finally, a multivariate non-linear decomposition analysis was employed to decompose rural-urban disparities in induced abortion. The multivariate non-linear decomposition analysis partitions disparity in induced abortion into components attributable to changing characteristics of women and due to changing reproductive behavior of the women. This technique also partitions the two components into segments that represent the contribution of each predictor to each of the two components (C and E) in a detailed decomposition. Component C refers to the part of change attributable to changing reproductive behavior while component E denotes disparity attributable to changing characteristics. To take care of the complex nature of the GMHS data, we used the “svyset” command during analysis, and weight and cluster were taken into consideration.

Results

Bivariate analysis of factors associated with induced abortion among women in Ghana

Table 1 presents the characteristics of the study population and factors associated with induced abortion. Most of the participants were aged 35-49 years (43.8%), 52.5% resided in urban areas

and more than half (57.8%) resided in the southern region of Ghana. More than a third of the women had Middle/JHS education (40.0%), close to half (48.7%) were currently married, and approximately 8 in 10 women were Christians (79.6%). A little over half experienced sexual debut before 18 years (55.9%), 70.2% were not currently using contraceptives, 59.4% had wrong knowledge of ovulation, 57.1% had a parity of 1-3 children, 9 in 10 women (90.6%) do not have knowledge of abortion legality and 62.6% were migrant. The prevalence of induced abortion in the study was 27.1%. Induced abortion was higher in urban areas (34.1%) than in rural areas (19.4%). Except for age, there were statistically significant differences in induced abortion across all the characteristics of women (Table 1). Regarding those residing in rural areas, except for migration status, there were statistically significant differences in induced abortion across all characteristics (Table 2). Except for age and knowledge of abortion legality, there were statistically significant differences in induced abortion across all characteristics in urban areas (Table 2).

Rural-urban disparities in factors associated with induced abortion among women in Ghana

The odds of induced abortion among women residing in the southern regions in both urban (AOR=2.88; 95%CI=2.37-3.52) and rural areas (AOR=3.10; 95%CI=2.51-3.85) were high compared to those residing in northern regions of Ghana (Table 3). Nevertheless, the odds were higher in rural areas. Compared with women with no education, women with any form of formal education were more likely to have had induced abortions in both urban and rural areas. However, the odds were higher among those with primary (AOR=1.78; 95%CI=1.45-2.18) or middle/JHS education (AOR=2.16; 95%CI=1.80-2.59) in urban areas. On the other hand, the odds of induced abortion were also higher among those with secondary (AOR=2.39; 95%CI=1.81-3.15) or higher education (AOR=2.29; 95%CI=1.49-3.53) in rural areas. The likelihood of induced abortion decreased with increasing parity in both rural (AOR=0.07; 95%CI=0.05-0.10) and urban areas (AOR=0.11; 95%CI=0.08-0.15). Overall, approximately 55% of the rural-urban disparities in induced abortion were attributable to differences in women's socio-demographic and obstetric characteristics (Table 4). Therefore, if women’s socio-demographic and obstetric characteristics were equaled, the rural-urban disparity in induced abortion would be decreased. Among the socio-demographic and obstetric characteristics, region of residence (25.4%), education (16.6%), and

parity (9.4%) explained approximately 51% of the rural-urban inequality in induced abortion (Table 5).

Table 1: Univariate and bivariate analysis of induced abortion among ever-pregnant women in Ghana, 2017

**Characteristics**	**Frequency**	**Percentage (%)**	**Induced abortion**		
			**No (%)**	**Yes (%)**	***p-value***
**Age**					0.282
15-24	3194	17.6	74.0	26.0	
25-34	6994	38.6	72.0	28.0	
35-49	7951	43.8	73.2	26.8	
**Place of Residence**					<0.001
Rural	8622	47.5	80.6	19.4	
Urban	9517	52.5	65.9	34.1	
**Region of Residence**					<0.001
Southern	10483	57.8	70.1	29.9	
Middle	5228	28.8	68.1	31.9	
Northern	2429	13.4	95.5	4.5	
**Educational Attainment**					<0.001
No education	4347	24.0	87.8	12.2	
Primary education	3147	17.4	73.4	26.3	
Middle/JHS	7251	40.0	66.9	33.1	
Secondary	2314	12.8	63.3	36.7	
Higher	1081	5.8	71.1	28.9	
**Marital Status**					
Currently married	8826	48.7	79.3	20.7	<0.001
Cohabiting	4977	27.4	68.9	31.1	
Not in union	4337	23.9	64.5	35.5	
**Religion**					
Catholic	1671	9.2	74.7	25.3	<0.001
Other Christians	12775	70.4	68.4	31.6	
Muslim	2786	15.4	87.7	12.5	
Traditionalist/Spiritualist	391	2.2	93.4	6.6	
No Religion	516	2.8	82.9	17.1	
**Current Contraceptive use**					<0.001
Yes	5405	29.8	69.9	30.1	
No	12735	70.2	76.7	23.3	
**Age at first Sex**					<0.001
< 18 years	10143	55.9	76.9	23.1	
18 years above	7997	44.1	67.1	32.9	
**Knowledge of Ovulation**					
Wrong Knowledge	10771	59.4			

Correct Knowledge	7369	40.6			
**Parity**					<0.001
No birth	1118	6.2	35.5	64.5	
1-3	10348	57.1	73.7	26.3	
4-5	4060	22.4	74.6	25.4	
6+	2613	14.4	83.2	16.8	
**Knowledge of Abortion Legality**					
Yes	1705	9.4	68.3	31.7	0.002
No	16435	90.6	73.4	26.6	
**Migration Status**					
Migrant	11358	62.6	71.4	28.6	<0.001
Non-Migrant	6782	37.4	75.5	24.5	
**Total**	18,140	100.0	72.9	27.1	

Table 2: Bivariate analysis of induced abortion among women in Ghana stratified by place of residence

**Characteristics**	**Rural (n=8622)**		**Urban (n=9518)**	
	**Induced abortion Yes (%)**	**P value**	**Induced abortion Yes (%)**	**P-value**
**Age**		0.049		0.732
15-24	14.6		28.5	
25-34	13.4		28.8	
35-49	12.4		28.0	
**Region of Residence**		<0.001		<0.001
Southern	22.9		33.8	
Middle	22.1		37.6	
Northern	4.0		8.3	
**Educational Attainment**		<0.001		<0.001
No education	5.5		12.5	
Primary education	14.2		28.3	
Middle/JHS	22.1		35.5	
Secondary	26.0		34.0	

Higher	23.4		23.1	
**Marital Status**		<0.001		<0.001
Currently married	8.0		20.3	
Cohabiting	21.1		38.1	
Not in union	21.8		37.9	
**Religion**		<0.001		<0.001
Catholic	10.2		28.6	
Other Christians	19.2		35.8	
Muslim	4.9		10.7	
Traditionalist/Spiritualist	3.1		11.1	
No Religion	8.6		22.1	
**Current contraceptive use**		<0.001		<0.001
Yes	17.5		32.2	
No	88.6		26.8	
**Age at first Sex**		<0.001		<0.001
< 18 years	14.7		34.5	
18 years above	10.7		22.5	
**Knowledge of Ovulation**		<0.001		<0.001
Wrong Knowledge	10.6		25.2	
Correct Knowledge	18.8		33.2	
**Parity**		<0.001		<0.001
No birth	54.7		66.2	
1-3	13.3		25.7	
4-5	12.4		26.9	
6+	7.9		20.5	
**Knowledge of Abortion Legality**		<0.001		0.263
Yes	18.2		29.8	
No	12.9		28.2	
**Migration Status**		0.684		<0.001
Migrant	13.3		30.4	
Non-Migrant	13.0		24.9	
**Total**	**19.4**		**34.1**	

Table 3: Multivariable regression analysis of factors associated with induced abortion among women in Ghana by place of residence

	**Pooled**	**Urban**	**Rural**
	aOR [95%CI]	aOR [95%CI]	aOR [95%CI]
Age			
15-24(ref)			
25-34	1.93[1.70-2.20]***	1.97[1.66-2.34]***	1.66[1.362.03]***
35-49	2.63[2.28-3.03]***	2.42[2.01-2.92]***	2.33[1.86-2.93]***
**Region**			
Northern (ref)			
Southern	3.27[2.84-3.77]***	2.88[2.37-3.52]***	3.10[2.51-3.85]***
Middle	3.59[3.08-4.18]***	3.53[2.87-4.35]***	2.92[2.31-3.69] ***
**Migration Status**			
Migrant (ref)			
Non-migrant	0.79[0.72-0.86]***	0.77[0.69-0.86]***	0.87[0.76-1.00]*
**Religion**			
Catholic (ref)			
Other Christian	1.13[0.99-1.29]	0.99[0.82-1.19]	1.21[0.99-1.49]
Islam	0.77[0.65-0.92]**	0.54[0.43-0.68]***	0.93[0.70-1.23]
Traditional/Spiritualist	0.45[0.28-0.71]**	0.60[0.26-1.39]	0.49[0.27- 0.86]*
No Religion	0.76[0.56-1.03]	0.64[0.41-1.01]	0.92[0.61-1.40]
**Parity**			
No child (ref)			
1-3	0.13[0.11-0.16]***	0.14[0.12-0.18]***	0.12[0.09-0.16]***
4-5	0.12[0.10-0.15]***	0.13[0.12-0.19] ***	0.10[0.08-0.14] ***
6 or more	0.08[0.06-0.10]***	0.11[0.08-0.15] ***	0.07[0.05-0.10] ***
**Knowledge of Ovulation**			
Wrong knowledge (ref)			
Correct Knowledge	1.35[1.24-1.47]***	1.31[1.18-1.46]***	1.44[1.26-1.65]***
**Legal Abortion**			
Yes (ref)			
No	0.83[0.72-0.95]**	0.88[0.75-1.03]	0.77[0.60-0.99]*
**Contraceptive Use**			
Yes (ref)			
No	0.73[0.67-0.80]***	0.77[0.68-0.86]***	0.66[0.57-0.76]***
**Marital Status**			
Currently married (ref)			
Cohabiting	1.45[1.31-1.61]***	1.53[1.33-1.76]***	1.46[1.24-1.72]***
Not in union	1.54[1.38-1.71]***	1.52[1.33-1.74] ***	1.43[1.19-1.72]***

**Age at First Sex**			
Below 18 years (ref)			
18 years above	0.49[0.45-0.54]***	0.45[0.40-0.50]***	0.54[0.47-0.63]***
**Education**			
No education (ref)			
Primary	1.77[1.53-2.04]***	1.78[1.45-2.18]***	1.52[1.23-1.87]***
Middle/JHS	2.22[1.95-2.53]***	2.16[1.80-2.59] ***	1.85[1.53-2.24] ***
Secondary	2.59[2.20-3.04]***	2.21[1.79-2.73] ***	2.39[1.81-3.15] ***
Higher	1.84[1.49-2.27]***	1.49[1.15-1.93] **	2.29[1.49-3.53]***

Note: p value < 0.05=*, p value <0.01**, p value <0.001***

Table 4 Overall decomposition of the change in induced abortion

Components	Coefficient	P-value	95%CI	Percent (%)
E	0.08	0.000	0.08-0.09	55.4
C	0.07	0.000	0.06-0.08	44.6
R	0.15	0.000	0.14-0.16	100.0

Table 5 Multivariate decomposition analysis of factors associated with induced abortion disparity between rural and urban residence

	**Due to differences in characteristics**			**Due to differences in coefficient**		
	Coefficient	P value	Percent	Coefficient	P value	Percent
**Age**						
15-24 (ref)						
25-35	0.00	<0.001	2.17	0.01	0.209	4.66
36-49	0.00	<0.001	0.95	0.00	0.797	1.24
**Region**						
Northern (ref)						
Southern	0.03	<0.001	16.62	0.00	0.615	-1.72
Middle	0.01	<0.001	8.78	0.00	0.254	2.54
**Migration Status**						
Migrant (ref)						
Non migrant	0.00	<0.001	1.46	-0.01	0.168	-3.98
**Religion**						
Catholic (ref)						
Other Christians	0.00	0.917	-0.1	-0.01	0.153	-7.8
Islam	0.00	<0.001	-1	-0.01	0.005	-9.41
Traditionalist/Spiritualist	0.00	0.224	1.97	0.00	0.687	0.78
No Religion	0.00	0.054	1.03	0.00	0.25	-1.16
**Parity**						
No child (ref)						
1-3	-0.04	<0.001	-26.32	0.01	0.248	7.03
4-5	0.01	<0.001	7.64	0.01	0.087	6.51
6 or more	0.04	<0.001	28.04	0.01	0.026	9
**Knowledge of Ovulation**						
Correct knowledge (ref)						
Wrong knowledge	0.00	<0.001	2.0	0.00	0.289	-2.18
**Knowledge of abortion legality**						
Yes (ref)						
No	0.00	0.122	0.75	0.01	0.361	9.63
**Current contraceptive use**						
Yes (ref)						

No	0.00	<0.001	-0.07	0.01	0.096	7.92
**Marital Status**						
Currently married (ref)						
Cohabiting	0.00	<0.001	-1.04	0.00	0.674	0.79
Not in union	0.01	<0.001	3.57	0.00	0.581	0.76
**Age at first sex**						
Below 18 years (ref)						
18 years above	-0.01	<0.001	-9.28	-0.01	0.055	-5.4
**Educational Attainment**						
No education (ref)						
Primary	0.00	<0.001	-1.1	0.00	0.302	2.03
Middle/JHS	0.01	<0.001	7.05	0.00	0.264	3.16
Secondary	0.01	<0.001	7.94	0.00	0.662	-0.35
Higher	0.00	0.002	2.75	0.00	0.09	-0.66

Discussion

The current study sought to examine rural-urban disparity in induced abortion in Ghana using a multivariate non-linear decomposition analysis. We found that the overall prevalence of induced abortion was 27.1%, which contradicts a previous study that reported an induced abortion of 6.0% in India [18]. In addition, the findings of this current study are inconsistent with other studies that reported an induced abortion rate of 10.1% in China [19], 13.6% in Ethiopia [20], and 16.0% in Nepal [21]. The high prevalence of induced abortion in this study could be because we limited the data to women who have had an abortion in their lifetime. Also, evidence shows that there has been an increase in contraceptive use in Ghana from 22% in 2014 to 25% in 2017% and an increase in the knowledge of the legality of abortion from 4% in 2007 to 11% in 2017 [22]. Probably, women tend to prevent unwanted pregnancies by using contraceptives, and those who get pregnant use legal means to abort a pregnancy by accessing safe abortion methods or they do not report induced abortion.

Our findings point to rural-urban differentials in induced abortion. Induced abortion was higher in urban areas (34.1%) than in rural areas (19.4%). The findings of this study corroborate other studies that reported a higher proportion of induced abortion in urban areas [18, 23]. The result is also inconsistent with previous studies that have reported that rural areas have a disproportionately higher prevalence of induced abortion than urban areas [15]. Evidence shows that in rural areas there is a lack of access to safe abortion health services or procedures. Also, the socio-economic conditions make it very difficult for some people to afford safe abortions. In addition, women in rural areas have a lower degree of autonomy and high unmet need which sometimes lead to unwanted pregnancy and consequently, induced abortion [15, 24]. The plausible reason why induced abortion is higher in urban areas than in rural areas could be attributed to high modern contraceptive use in rural areas (27%) compared with 23% in urban areas. Also, high unmet needs in urban areas [22], and the fear of the stigma of being pregnant are mostly for unmarried women. Also, the socio-economic conditions such as the high cost of living in the urban areas compared to the rural areas could contribute to the high induced abortion in urban areas.

Consistent with the findings of a related study by Wei et al. [25], rural-urban disparities in induced abortion were attributable to the differences in parity. The regression analyses show that the odds of induced abortion significantly declined with an increase in parity. This implies that women

without a child were more likely to have induced abortions than those with a child (ren). As the number of children increases, the odds of inducing abortion decrease. The situation is the same for the pooled data as well as the rural-urban data. However, the odds were much lower for those in rural areas than those in urban areas. Evidence indicates that socioeconomic factors, especially, education, marital status, and fear of stigma have been identified as some of the reasons why women without a child may induce pregnancy more than those with a child(ren) [26]. Due to stigma or shame, unmarried women may postpone childbirth by inducing a pregnancy. Culturally, postponing childbearing to a more suitable time, especially after marrying is like a norm that regulates the behavior of both rural and urban residents in Ghana [27, 28]. In addition, unwanted pregnancy and restriction of family size could explain a decrease in the odds of induced abortion when parity increases.

Education of women emerged as an important factor that explains rural-urban differentials in induced abortion. Greater odds were reported for women with education. Educated women were more likely to induce abortion at all levels than those without education. This was similar for those in rural and urban areas. The results agree with other studies [29, 30]. Prior studies reported that education exposes people to many things, including higher knowledge of healthcare, and abortion laws, as well as enabling women to have greater autonomy in their decisions [31, 32, 33]. The abortion laws in Ghana frown on the termination of pregnancy. However, a pregnancy can be terminated based on certain factors such as rape and health conditions [31]. The probable reason why educated women in this study induce abortion could be attributed to their knowledge and awareness of the abortion laws, which could influence their decision to induce a pregnancy rather than look for a safe abortion process. In this current study, the odds of induced abortion were higher for women with Middle or Second-cycle education in both the rural and urban areas. Consequently, women in school especially in the middle, secondary, and tertiary may recourse to induced abortion to prevent unwanted pregnancies to enable them to further their education. Biggs et al. [32] argued that women perceived the difficulty of schooling and achieving their career goals when raising a child and this sometimes leads to induced abortion.

We also found that the place of region explains the rural-urban differentials in induced abortion. The results show that women in the Southern and Middle regions were more likely to induce a pregnancy than women in the northern region. Contextually, both the middle and southern regions

are more developed than the northern regions. Hence, most of the residents may have autonomy and the socio-economic conditions may influence the decisions of women to induce a pregnancy.

Strengths and limitations of the study

The GDHS data is nationally representative and this makes it possible for our results to be generalized. However, our data was limited to women who had induced abortions in their lifetime. Hence, the results should be interpreted in line with lifetime induced abortion other than only induced abortion within five years. Again, GDHS is a cross-sectional study that cannot be used to establish causality, but rather, an association (s) between variables. In addition, the self-report measure of the outcome variable, induced abortion, has the potential for unintentional bias.

Conclusion and Recommendation

We found a significant disparity in induced abortion in urban and rural areas. The findings from this study suggest that women in urban areas have more induced abortions than women in rural areas. The difference in the rural-urban disparity is attributed to parity, education level of women, and region of residence. Policies should target women with formal education in both rural and urban areas aiming to prevent induced abortion. Women should be encouraged to use safe abortion procedures such as accessing health care for abortion services. In addition, women in urban areas should be encouraged to use contraceptives to prevent unwanted pregnancies. There should also be education on the abortion process as well as easy accessibility of abortion services for both women in rural and urban areas to reduce induced abortion.

List of abbreviations

AOR	Adjusted Odds Ratio
GDHS	Ghana Demographic and Health Survey
GHS	Ghana Health Service
GMHS	Ghana Maternal Health Survey
GSS	Ghana Statistical Service
ICPD	International Conference on Population and Development
NMIMR	Noguchi Memorial Institute for Medical Research
SDG	Sustainable Development Goal
UN	United Nations
WHO	World Health Organization

Acknowledgments
Not applicable

Author Contributions

IY led the conceptualization and analysis of the research, MWA and MNE participated in the process. IY and MWA participated in drafting the manuscript. DD, DK, AKC and SAOK reviewed and edited the draft. All authors reviewed the manuscript.

Data Availability

The datasets generated and analyzed for this study are available in the MEASURE DHS database at the repository; https://dhsprogram.com/data/dataset/Ghana_Special_2017.cfm?flag=1

Ethical approval

The 2017 Ghana Maternal Health Survey was conducted under the scientific guidance of the Ghana Statistical Service, Ghana Health Service (GHS), and Noguchi Memorial Institute for Medical Research (NMIMR). ICF International approved the survey and provided technical assistance. Written Informed consent was obtained from all the respondents before the commencement of interviews with each interviewer.

Declaration of Conflicting interest

The author(s) declared no potential conflicts of interest with respect to the research, authorship, and/or publication of this article.

Funding

This research did not receive any specific grant from funding agencies in the public, commercial, or not-for-profit sectors

References

1. World Health Organization (WHO). Preventing unsafe abortion [Internet]. 2019 [cited 2022 Oct 27]. Available from: https://www.who.int/news-room/fact-sheets/detail/preventing-unsafe-abortion.
2. Shah IH, Åhman E, Ortayli N. Access to safe abortion: progress and challenges since the 1994 International Conference on Population and Development (ICPD). Contraception. 2014;90(6):S39-S48.
3. Awowole IO, Ijarotimi OA. Restrictive abortion laws, COVID-19, telehealth, and medication abortion in the SDG era. Lancet Glob Health. 2022;10(1):e14-e15.
4. World Health Organization (WHO). Abortion: key facts [Internet]. 2020 Nov [cited 2022 Oct 27]. Available from: https://www.who.int/news-room/fact-sheets/detail/abortion.
5. Bearak J, Popinchalk A, Ganatra B, Moller AB, Tunçalp Ö, Beavin C, et al. Unintended pregnancy and abortion by income, region, and the legal status of abortion: estimates from a comprehensive model for 1990–2019. Lancet Glob Health. 2020;8(9):e1152-e1161.
6. Say L, Chou D, Gemmill A, Tunçalp Ö, Moller AB, Daniels J, et al. Global causes of maternal death: a WHO systematic analysis. Lancet Glob Health. 2014;2(6):e323-e333.
7. Klu D, Yeboah I, Kayi EA, Okyere J, Essiaw MN. Utilization of abortion services from an unsafe provider and associated factors among women with history of induced abortion in Ghana. BMC Pregnancy Childbirth. 2022;22(1):1-8.
8. Ngwena C. Access to Safe Abortion as a Human Right in the African Region: Lessons from Emerging Jurisprudence of UN Treaty monitoring Bodies. S Afr J Hum Rights. 2013;29(2):399-428.
9. Yokoe R, Rowe R, Choudhury SS, Rani A, Zahir F, Nair M. Unsafe abortion and abortion-related death among 1.8 million women in India. BMJ Glob Health. 2019;4(3):e001491
10. Dasgupta P, Biswas R, Das DK, Roy JK. Pro-life or pro-abortion–Women's attitude toward abortion in Darjeeling, India. Arch Med Health Sci. 2019;7(1):42.
11. Danso NA, Nagumsi BAA, Twi-Yeboah A, Boamah FA, Cadri A. The rate of abortion in Ghana among women of reproductive age: Analysis of the 2007 and 2017 Maternal Health Surveys. Ethics Med Public Health. 2022;22:100769.

12. Amo-Adjei J, Darteh EK. Unmet/met need for contraception and self-reported abortion in Ghana. Sex Reprod Healthc. 2017;13:118-124.
13. Chae S, Desai S, Crowell M, Sedgh G, Singh S. Characteristics of women obtaining induced abortions in selected low-and middle-income countries. PLoS One. 2017;12(3):e0172976.
14. Huang D, Ngai SSY. Decision-making, abortion narratives and identity construction towards abortion among rural–urban single migrant women in mainland China: Insights for social work practice and education. Int Soc Work. 2022;65(5):870-882.
15. Rahaman M, Das P, Chouhan P, Das KC. Examining the rural-urban divide in predisposing, enabling, and need factors of unsafe abortion in India using Anderson's behavioral model.
16. Hoffman LW. Hoffman Developmental Psychology Today. Europe: McGraw-Hill Education; 1993.
17. Bandura A. Self-efficacy: toward a unifying theory of behavioral change. Psychol Rev. 1977;84(2):191.
18. Elul B, Barge S, Verma S, Kumar N, Bracken HJ, Sadhwani H. Unwanted pregnancy and induced abortion: data from men and women in Rajasthan, India.
19. Wang X, Wu J, Li Y, Zhou Y, Li Y, Zhao R, et al. Changes in the prevalence of induced abortion in the floating population in major cities of China 2007–2014. Int J Environ Res Public Health. 2019;16(18):3305.
20. Lentiro K, Gebru T, Worku A, Asfaw A, Gebremariam T, Tesfaye A. Risk factors of induced abortion among preparatory school student in Guraghe zone, Southern region, Ethiopia: a cross-sectional study. BMC Womens Health. 2019;19(1):1-7.
21. Yogi A, KC P, Neupane S. Prevalence and factors associated with abortion and unsafe abortion in Nepal: a nationwide cross-sectional study. BMC Pregnancy Childbirth. 2018;18(1):1-10.
22. Ghana Statistical Service (GSS), Ghana Health Service (GHS) and Macro ICF. Ghana Maternal Health Survey 2017. Accra: Ghana Statistical Service; 2018.
23. Ratovoson R, Kunkel A, Rakotovao JP, Pourette D, Mattern C, Andriamiadana J, et al. Frequency, risk factors, and complications of induced abortion in ten districts of Madagascar: results from a cross-sectional household survey. BMC Womens Health. 2020;20(1):1-11.
24. Banerjee SK, Andersen K. Exploring the pathways of unsafe abortion in Madhya Pradesh, India. Glob Public Health. 2012;7(8):882-896.
25. Wei Z, Yu D, Liu H. Induced abortion among married women in China: a study based on the 1997–2017 China Fertility Surveys. China Popul Dev Stud. 2021;5(1):83-99.
26. Agula C, Henry EG, Asuming PO, Agyei-Asabere C, Kushitor M, Canning D, et al. Methods women use for induced abortion and sources of services: insights from poor urban settlements of Accra, Ghana. BMC Womens Health. 2021;21(1):1-12.

27. Ameyaw EK. Prevalence and correlates of unintended pregnancy in Ghana: Analysis of 2014 Ghana Demographic and Health Survey. Matern Health Neonatol Perinatol. 2018;4(1):1-6.
28. Eliason S, Baiden F, Yankey BA, Awusabo-Asare K. Determinants of unintended pregnancies in rural Ghana. BMC Pregnancy Childbirth. 2014;14(1):1-9.
29. Lamina MA. Prevalence of abortion and contraceptive practice among women seeking repeat induced abortion in Western Nigeria. J Pregnancy. 2015;2015:486203.
30. Senbeto E, Alene GD, Abesno N, Yeneneh H. Prevalence and associated risk factors of induced abortion in Northwest Ethiopia. Ethiop J Health Dev. 2005;19(1):37-44.
31. Assifi AR, Berger B, Tunçalp Ö, Khosla R, Ganatra B. Women's awareness and knowledge of abortion laws: A systematic review. PLoS One. 2016;11(3):e0152224.
32. Biggs MA, Gould H, Foster DG. Understanding why women seek abortions in the US. BMC Womens Health. 2013;13(1):1-13.
33. Konney TO, Danso KA, Odoi AT, Opare-Addo HS, Morhe ES. Attitude of women with abortion-related complications toward provision of safe abortion services in Ghana. J Womens Health (Larchmt). 2009;18(11):1863-1866.
