# Supplementary material for: Rural-urban disparity in induced abortion in Ghana: A multivariate non-linear decomposition analysis of the Ghana Maternal Health Survey
Source: PLoS One. 2025 Oct 15;20(10):e0334024. doi: 10.1371/journal.pone.0334024 (PMC12527179; doi:10.1371/journal.pone.0334024)
Supplement: Attachment — Submitted filename: Review Comments_Plos.docx [file pone.0334024.s001.docx]

Thank you so much for providing me the opportunity to review the manuscript entitled "Rural-urban disparity in induced abortion in Ghana: a multivariate non-linear decomposition analysis of Ghana Maternal Health Survey". The manuscript meets all the scientific standards necessary to be published. However, the paper has some minor flaws which need to be addressed before it is suitable for publication.

1. In the abstract section, the same phrase has been repeated. Kindly remove the repeated one.

2. In the multivariate decomposition table, there is no hyphen between the lower and upper limits of CI. Kindly rectify the same.

3. There are a number of variables with missing responses. But you have not mentioned whether you have accommodated missing responses or excluded the same. So, address the same in your methodology section in nutshell.

4. You have put forth an insightful implication of Social Learning Theory. But you have not mentioned is there any specific model which you have used for executing the role of the aforementioned theory on rural urban divide in induced abortion. Kindly elaborate in your methodology section.

5. You have mentioned that you have chosen potential confounders based on prior literature although there is no citation for the same. Please make sure proper citation of the previous studies.

6. Please revise the **Table 1** as there is no data for the variable called ‘Knowledge of ovulation’. It seems like you have overlooked the same. If that is not the case please make sure to add a note for the same.

7. Please try to stick on same categories of the variables throughout your writing. For instance, somewhere you have used mathematical sign for parity (6+) nevertheless have used text for the same (6 or more).
